# Extended local similarity analysis (eLSA) of microbial community and other time series data with replicates

**DOI:** 10.1186/1752-0509-5-S2-S15

**Published:** 2011-12-14

**Authors:** Li C Xia, Joshua A Steele, Jacob A Cram, Zoe G Cardon, Sheri L Simmons, Joseph J Vallino, Jed A Fuhrman, Fengzhu Sun

**Affiliations:** 1 Molecular and Computational Biology Program, Department of Biological Sciences, University of Southern California, Los Angeles, CA 90089-2910, USA; 2 Division of Geological and Planetary Sciences, California Institute of Technology, Pasadena, CA 91125, USA; 3 Marine and Environmental Biology, Department of Biological Sciences, University of Southern California, Los Angeles, CA 90089-0371, USA; 4 The Ecosystems Center, Marine Biological Laboratory, Woods Hole, MA 02543, USA; 5 Bay Paul Center, Marine Biological Laboratory, Woods Hole, MA 02543, USA

## Abstract

**Background:**

The increasing availability of time series microbial community data from metagenomics and other molecular biological studies has enabled the analysis of large-scale microbial co-occurrence and association networks. Among the many analytical techniques available, the Local Similarity Analysis (LSA) method is unique in that it captures local and potentially time-delayed co-occurrence and association patterns in time series data that cannot otherwise be identified by ordinary correlation analysis. However LSA, as originally developed, does not consider time series data with replicates, which hinders the full exploitation of available information. With replicates, it is possible to understand the variability of local similarity (LS) score and to obtain its confidence interval.

**Results:**

We extended our LSA technique to time series data with replicates and termed it extended LSA, or eLSA. Simulations showed the capability of eLSA to capture subinterval and time-delayed associations. We implemented the eLSA technique into an easy-to-use analytic software package. The software pipeline integrates data normalization, statistical correlation calculation, statistical significance evaluation, and association network construction steps. We applied the eLSA technique to microbial community and gene expression datasets, where unique time-dependent associations were identified.

**Conclusions:**

The extended LSA analysis technique was demonstrated to reveal statistically significant local and potentially time-delayed association patterns in replicated time series data beyond that of ordinary correlation analysis. These statistically significant associations can provide insights to the real dynamics of biological systems. The newly designed eLSA software efficiently streamlines the analysis and is freely available from the eLSA homepage, which can be accessed at http://meta.usc.edu/softs/lsa.

## Background

In recent years, advances in microbial molecular technologies, such as next generation sequencing and molecular profiling, have enabled researchers to spatially and temporally characterize natural microbial communities without laboratory cultivation [1]. However, to reveal existing symbiotic relationships and microbe-environment interactions, it is necessary to mine and analyze temporal and spatial co-occurrence association patterns of organisms within these new datasets [2,3]. Time series data, in particular, are receiving increased attention, since not only ordinary associations, but also other local and potentially time-delayed associations can be inferred from these datasets. Here local association indicates that the association only occurs in a subinterval of the time of interest, and time-delayed association indicates that there is a time lag for the response of one organism to the change in another organism. The rapid accrual of time series data is not limited to the microbial ecology field. Progress in high-throughput low-cost experimental technologies has also brought such changes to gene transcription and translation studies. Thus, while the subjects may vary, the association network we build from local and potentially time-delayed association patterns will likely pave the way to a better understanding of these systems.

To analyze microbial community and other data under various conditions, researchers typically use techniques such as Pearson’s Correlation Coefficient (PCC), principal component analysis (PCA), multi-dimensional scaling (MDS), discriminant function analysis (DFA) and canonical correlation analysis (CCA) [4-8]. Although these analytic methods yield interesting patterns, they generally analyze the data throughout the whole time interval of interest without considering potential local and time-delayed associations. We are specifically interested in discovering local and potentially time-delayed associations. Such associations have been shown to play important roles in understanding gene expression dynamics and the association of organisms in microbial communities [9-12].

To understand local and time-delayed associations, we originally designed a Local Similarity Analysis (LSA) for time series data measured typically at successive and equal time intervals without replicates [11]. Studies adopting the original LSA technique have shown interesting and novel discoveries for microbial community datasets. To name a few, Paver et al. [10] successfully applied LSA to study glycolate-utilizing bacterial and phytoplankton associations, while Shade et al. [13] used LSA to discover bacterial association dynamics during lake mixing.

Since biological experiments are often associated with many potential sources of noise, repeated measurements (replicates) are usually carried out in order to better assess inherent uncertainties of the quantities of interest [14]. Furthermore, data emerging from such experiments are typically analyzed by mean effect or by the development of profiles where variability is not properly accounted for [15]. Temporal and spatial data with replicates are being generated in Dr. Cardon’s laboratory and others. The lack of support for replicated data in the original LSA program has prevented its application to these new datasets. With replicates, it is possible to evaluate the variation of and to give a bootstrap confidence interval for the local similarity (LS) score as defined in *Ruan et al.*[11]. Furthermore, the original LSA is restricted by the low computing efficiency of the R language, as well as poor handling of missing values. In order to improve upon these issues and make the technique more accessible to the scientific community, we developed an extended LSA technique, named eLSA, and implemented it as a C++ extension to Python.

Briefly, given time series data of two factors and a user-constrained delay limit, eLSA finds the configuration of the data that yields the highest local similarity (LS) score, which is a type of similarity metric. For example, within a delay limit of two units, the first time spot of one series might be aligned to the third time spot of the other series, thus maximizing their LS. For a dataset of many factors, eLSA is applied to each pairwise combination of factors in the dataset. Candidate associations are then evaluated statistically by a permutation test, which calculates the p-value which is the proportion of scores exceeding the original LS score after shuffling the first series and re-evaluating the LS score many times, and by the false discovery rate (FDR q-value), which is used to correct multiple comparisons. Researchers can use eLSA to detect undirected associations, i.e., association patterns without time delays, and directed associations, where the change of one factor may temporally lead or follow another factor.

The organization of the paper is as follows. In the “Methods” section, we describe the LSA algorithm for calculating LS score with replicates, data normalization, estimation of confidence interval for the LS score, and testing the statistical significance of a LS score. In the “Results” section, we first show the efficacy of eLSA by simulations, then describe briefly the pipeline of eLSA, and finally apply the pipeline to analyze a microbiological dataset and a gene expression dataset. The paper concludes with some discussion and conclusions.

## Methods

### Pearson’s correlation coefficient-based analysis

Suppose that the time series data for factors *X* and *Y* with replicates are measured simultaneously. We denote them as *X* = *X*_[1:*n*][1:*m*]_ and *Y* = *Y*_[1:*n*][1:*m*]_, where *n* is the number of samples (time points) and *m* is the number of replicates. Let *X_i_*_[1:*m*]_ and *Y_j_*_[1:*m*]_, or, in more abbreviated form, *X_i_* and *Y_j_*, be the vectors containing the *m* replicates from the *i*-th time spot of *X* and the *j*-th time spot of *Y*, respectively. The application of Pearson’s Correlation Coefficient (PCC) requires taking the profile means, *i.e.* and . Then the PCC between *X* and *Y* is defined as:(1)

where , ,  and  are the means of *X* and *Y*, respectively. The statistical significance of *r* is tested by the fact that  follows a *t*-distribution (degree of freedom: *v* = *n* – 2, mean: 0 and variance *v* / (*v* – 2)) when *m* = 1. For a pair of non-replicated series where *m* = 1, PCC is a straightforward and powerful method to test and identify linear relationship between two bivariate normally distributed random variables. It is widely adopted in the literature but with limitations. Specifically, when the real relationships are more complex, for example, the association between the two factors only occurs in a subinterval of the region of interest or the change of one factor has a time-delay in response to the change of another factor. Several methods, including the original LSA method, have been proposed to overcome such difficulties [11,16].

### Local similarity analysis with replicates

The original LSA method considers only data without replicates. In this paper, we extend the Local Similarity Analysis (LSA) method [11] to samples with replicates. To formulate the algorithm, we suppose each sample have *m* replicates and let *F*(·) be some summarizing function for the repeated measurements. Thus, we extend the original LSA dynamic programming algorithm to data with replicates as follows:

(1) For *i*, *j* in {1,2,…,*n*}^2^:

*P*_0,*j*_ = 0, *P*_*i*,0_ = 0, and *N*_0,*j*_ = 0, *N*_*i*,0_ = 0.

(2) For *i*, *j* in {1,2,…,*n*}^2^ with |*i* – *j*|≤ *D*:

*P*_*i*+1,*j*+1_ = max{0,*P*_*i*,*j*_ + *S_XY_*[*F*(*X_i_*),*F*(*Y_j_*)]} and

*N*_*i*+1,*j*+1_ = max{0,*N*_*i*,*j*_ + *S_XY_*[*F*(*X_i_*),*F*(*Y_j_*)]}.

(3) *P_max_*(*X*,*Y*) = max_1≤*i*,*j*≤*n*_*P*_*i*,*j*_ and

*N_max_*(*X*,*Y*) = max_1≤*i*,*j*≤*n*_*N*_*i*,*j*_.

(4)  and

*S_sgn_*(*X*,*Y*) = sgn[*P_max_*(*X*,*Y*) – *N_max_*(*X*,*Y*)].

The *S_max_*(*X*,*Y*) obtained is the maximum local similarity score possible for all configurations of *m*-replicated time series *X* and *Y* within time-delay *D*. In this extended algorithm, the scalars *x_i_* ’s and *y_i_* ’ s from the non-replicated series in *Ruan et al.*[11] are replaced by vector functions *F*(*X_i_*)’s and *F*(*Y_j_*)’s to handle data with replicates. Alternatively, we can also consider *F*(*X_i_*)’s and *F*(*Y_j_*)’s as the same input data for the original algorithm in *Ruan et al.*[11], except that they are *F*-transformed data. In addition, this extended LSA framework easily accommodates the original version of LSA without replicates using *m* = 1 as a special case.

### Different ways of summarizing the replicate data

Notice that the only additional component we introduced in the eLSA algorithm is the function *F*. Many reports have suggested different possible forms for *F*, and several computational methods have been proposed for summarizing the additional information available from replicates, including the simple average method (abbreviated as ‘simple’) and the Standard Deviation (SD)-weighted average method (abbreviated as ‘SD’), and the multivariate correlation coefficient method [17-19]. However, the result of the multivariate correlation coefficient method from *Zhu et al.*[17] can be shown to be the same as the ‘simple’ method. Therefore, in eLSA, we used the first two methods. We also propose the use of median in place of average and Median Absolute Deviation (MAD) in place of SD when robust statistics are needed to handle outliers [20]. The corresponding methods are named simple median method (abbreviated as ‘Med’) and MAD-weighted median method (abbreviated as ‘MAD’), respectively.

The ‘simple’ method is, in spirit, to take the mean profiles to represent the replicated series. In practice, we take *F* to be the simple average of repeated measurements: . The ‘SD’ method, on the other hand, takes the standard deviation of the replicates into account. Here we take *F* to be the replicate average divided by its standard deviation (SD): . Importantly, this method utilizes the variability information available, and, as such, it is claimed to be better than the ‘simple’ method in estimating the true correlation [18]. However, in order for the ‘SD’ method to be effective, a relatively large number of replicates, *m*, are needed, *e.g.,m* ≥ 5. For a small number of replicates, the ‘SD’ method may not work well since the standard deviation may not be reliably estimated. Further, if we replace average with median and SD with MAD, we obtain the ‘Med’ method: *F*(*X_i_*) = *Median*(*X_i_*) and the ‘MAD’ method: , where *MAD*(*X_i_*) = *Median*(|*X_i_* – *Median*(*X_i_*)|). The two transformations have similar properties as their corresponding average and SD versions, but they are more robust.

### Bootstrap confidence interval for the LS score

With replicate data, researchers can study the variation of quantities of interest and to give their confidence intervals. Due to the complexity of calculating the LS score, the probability distribution of the LS score is hard to study theoretically. Thus, we resort to bootstrap to give a bootstrap confidence interval (CI) for the LS score. Bootstrap is a re-sampling method for studying the variation of an estimated quantity based on available sample data [21]. In this study, we use bootstrap to estimate a confidence interval for the LS score. For a given type I error α, the 1 – α confidence interval is the estimated range that covers the true value with probability 1 – α. Thus, for a given number, *B*, of bootstraps, we construct the bootstrap sample set , where each  and  are samples with replacement from *X_i_* and *Y_j_*, respectively. The rest of the calculation is the same as that used for the original data, and we obtain . Without the loss of generality, we suppose that these values are sorted in ascending order: . Then, a 1 – α bootstrap CI of *S_max_* can be estimated by , as suggested by *Efron et al.*[21].

### Data normalization

eLSA analyses require the series of factors *X* and *Y* to be normally distributed, but this may not be the case in the real dataset. Therefore, through normalization, the normality of the data can be enforced. To accommodate possible nonlinear associations and the variation of scales within the raw data, we apply the following approach [22] to normalize the raw dataset before any LS score calculations. We use *F*(*X_i_*) to denote the *F*-transformed data of the *i*-th time spot of an variable *X_i_*. First, we take (2)

Then, we take (3)

where Φ is the cumulative distribution function of the standard normal distribution. We will take *Z* = *Z*_[1:*n*]_ obtained through the above procedure as the normalization of *X*. Therefore, the normalization steps are taken after the *F*-transformation.

### Permutation test to evaluate the statistical significance of LSA association

It is important to evaluate the statistical significance of the LS score measured by the p-value, the probability of observing a LS score no smaller than the observed score when two factors are not associated locally or globally. To achieve this objective, permutation test is used. To perform the test, we fix *Y* and reshuffle all the columns of *X* for each permutation. For a fixed number of permutations *L*, suppose {*X*^(1)^,*X*^(2)^,…,*X*^(*L*)^} is the permuted set of *X*; then the p-value *P_L_* is obtained using(4)

where *I*(·) is the indicator function. With large enough number of permutations, we can evaluate the p-value to any desired accuracy.

### False discovery rate (FDR) estimation

In most biological studies, a large number of factors need to be considered. If there are *T* factors, there will be  eLSA pairwise calculations, representing its quadratic growth in *T*. In order to avoid many falsely declared associated pairs of factors, we need to correct for multiple testing. Many methods have been developed to correct for multiple testing and here we use the method by *Storey et al.*[23] to address this issue. In particular, we report the q-value, *Q*, for each pair of factors. The q-value for a pair of factors is the proportion of false positives incurred when that particular pair of factors is declared significant.

### Computation complexity and implementation

For a single pair of time series, the time complexity for calculating the LS score using the dynamic programming algorithm is *O*(*n*), where *n* is the number of time points. The estimation of the bootstrap confidence interval for the LS score using *B* bootstraps will need *O*(*Bn*) calculations. The estimation of statistical significance for each pair of factors using *L* permutations will need *O*(*Ln*) calculations. Thus, the number of calculations for a full analysis of each pair of factors will be *O*(*BLn*). If there are a total of *T* factors, there are a total of  pairs of factors that need to be compared. Thus, the number of calculations for a full analysis of *T* factors will be in the order of *O*(*T*^2^*BLn*), which can be computationally intensive.

In summary, the internal support for replicates and the use of CI estimates are the two major methodological enhancements to LSA. The eLSA software, however, also incorporates other new features, such as faster permutation and false discovery rate evaluations and more options to handle missing values. Other implementation details are available from the software documentation.

## Results

### Simulations and benchmarks

We generated simulated data to show the efficacy of eLSA in capturing time-dependent association patterns, such as time-delayed associations and associations within a subinterval. We also studied the difference between the eLSA inference using the simple average (referred to as ‘simple’) method, the SD-weighted average method (referred to as ‘SD’), the median (referred to as “Med”) method, and the MAD (referred to as ‘MAD’) method.

#### Time-delayed association

**Figure 1:**
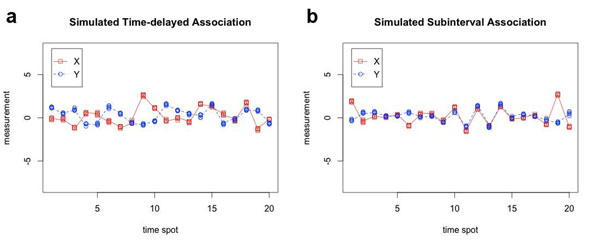
**Examples of simulated associations.** a. An example of simulated time-delayed association series with five replicates is shown, where X (red square) leads Y (blue circle) by three time units. The pattern is not significant by ordinary correlation analysis (PCC=-0.258, P=0.272); however, it is captured by local similarity analysis (LS=0.507, P=0.006). b. An example of simulated subinterval association series with five replicates is shown, where X (red square) and Y (blue circle) are associated in the time interval from 6 to 15. The pattern is not significant by ordinary correlation analysis (PCC=0.258, P=0.273); however, it is captured by local similarity analysis (LS=0.428, P=0.028).

In this case, *X* and *Y* are assumed to be positively correlated with a time delay *D*. For a particular example with *D* = 3, we assume that (*X*_*j*+3_,*Y_j_*)’s follows a bivariate normal distribution with mean *μ* = **0** and covariance matrix , for *j* = 1,2,…,20, where *ρ* = 0.8. *X_j_*’s are assumed to be standard normal for *j* = 1,2,3. The generated (*X_j_*,*Y_j_*)’s are further perturbed *m* times by a measurement disturbance *ε_ij_* : *N*(0, 0.01) to obtain the *m*-replicated series. A pair of simulated series is shown in Figure 1a for a typical simulation with *m* = 5.

We see that the two series closely follows each other if we shift the *Y* series three units toward right. In this particular example, the PCC is -0.258 (*P*=0.272) while the LS score using ‘simple’ averaging method is 0.507 with a p-value of 0.006. We did 1000 bootstraps and the 95% bootstrap confidence interval for this particular example is (0.448, 0.549). Therefore, this time-delayed association is only found significant by the eLSA analysis.

#### Association within a subinterval

In this case, we assume *X* and *Y* are positively associated within a subinterval and not associated in other regions. In our simulation, we generate 20 time spots of the two series by sampling (*X_j_*,*Y_j_*) from a bivariate-normal distribution with mean *μ* = **0** and covariance matrix  where *ρ* = 0.8 for 6 ≤ *j* ≤ 15, and *ρ* = 0 for *j* ≤ 5 or 16 ≤ *j* ≤ 20. The generated (*X_j_*,*Y_j_*)’s are further perturbed *m* times by a measurement disturbance *ε_ij_* : *N*(0, 0.01) to obtain the *m*-replicated series. One generated series are shown in Figure 1b for a typical simulation with *m* = 5.

We can see the two series mostly closely follow each other within the intended subinterval 6 ≤ *j* ≤ 15. In this particular example, the PCC is 0.258 (*P*=0.272) while the LS score using ‘simple’ averaging method is 0.428 with a p-value of 0.028. We did 1000 bootstraps and the 95% bootstrap confidence interval is (0.404, 0.446). This pattern is again uniquely captured by the eLSA analysis. In real applications, there are many other possibilities that two factors are associated without a significant Pearson or Spearman’s correlation coefficient. The eLSA can capture these associations as long as their LS score can be maximized through dynamically enumerating their configurations.

#### Different summarizing function

To see the effect of replicates, we also let *m* = 1, 10, 15, 20 in the time-delayed simulation and did the same analysis as above with 1000 simulations. The results are summarized in Table 1. It can be seen from the table that the results using “simple” and “Med” are similar with mean LS scores ranging from 0.490 to 0.498 and standard errors ranging from 0.078 to 0.091. On the other hand, if the noise in the replicates is not normally distributed, the “Med” method should be more robust. On the other hand, the mean LS scores using “SD” and “MAD” are generally lower than that using the “simple” and “Med” methods. This maybe caused by the extra variation introduced when estimating the standard deviation or maximum absolute deviation from the data.

**Table 1:** Mean and standard error of the estimated LS score

	m=1		m=5		m=10		m=15		m=20	
F-function	mean	se.	mean	se.	mean	se.	mean	se.	mean	se.
‘simple’	.495	.078	.495	.085	.491	.088	.493	.076	.496	.091
‘SD’	na.	na.	.332	.127	.391	.124	.412	.119	.435	.109
‘Med’	.495	.078	.490	.090	.490	.090	.490	.083	.498	.083
‘MAD’	na.	na.	.494	.115	.302	.128	.325	.129	.371	.119

The values are calculated based on 1000 simulations. ‘se.’ indicates standard error and ‘na.’ indicates not applicable.

#### Running time comparison

We benchmarked the running time performance of the new eLSA implementation and the old R script. For a dataset of 72 time series each with 35 time points, we tried eLSA analysis with 100 bootstraps, 1000 permutations and a delay limit of 3. It took the old script 20462 seconds to finish the computation while the new C++ program used 2054 seconds, which is about 9 times faster. Meanwhile, the new implementation also reduces the memory consumption and increases input/output efficiency. The benchmark is carried out on a “Dell, PE1950, Xeon E5420, 2.5GHz, 12010MB RAM” computing node.

### The eLSA analysis pipeline

**Figure 2:**
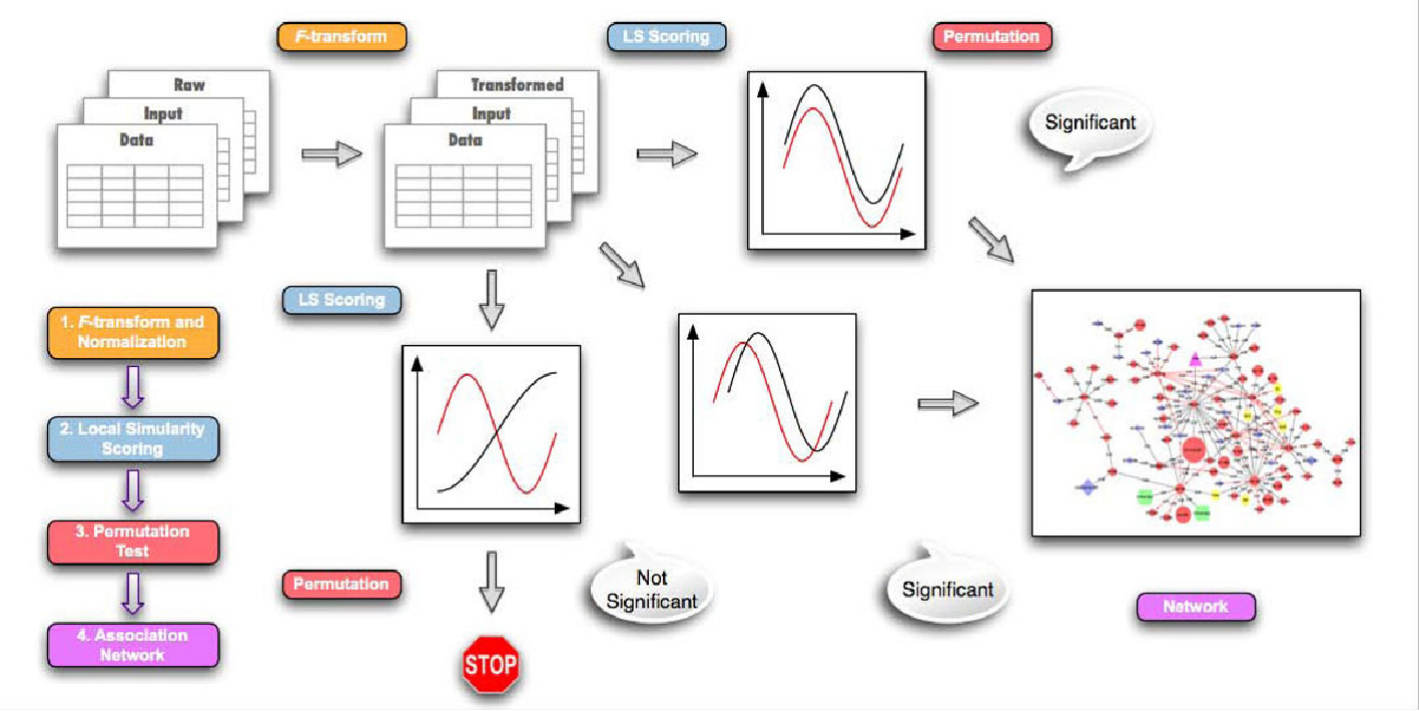
**eLSA pipeline**. Users start with raw data (matrices of time series) as input and specify their requirements as parameters. The LSA tools subsequently *F*-transform and normalize the raw data and calculate Local Similarity (LS) scores and Pearson’s Correlation Coefficients. The tools then assess the statistical significance (P-values) of these correlation statistics using the permutation test and filter out insignificant results. Finally, the tools construct a partially directed association network from the significant associations.

In this subsection, we briefly describe the eLSA analysis pipeline implemented into the eLSA software package, as shown in Figure 2.

#### F-transformation and data normalization

The eLSA tool accepts a matrix file where each row is a time series for one factor. It fills up missing data by a user-specified method. Zero to third order spline-based methods and the nearest neighbour method as implemented in the *Scipy* (http://www.scipy.org) interpolation module are available. It then transforms the data by the user-specified *F* function and normalizes the *F*-transformed data by the normal score transformation following *Li et al.*[22] (see Methods).

#### Local similarity scoring

Local similarity analysis calculates the highest similarity score between any pair of factors. Users can specify parameters, including, for example, the maximum shifts allowed. Local Similarity score is calculated using the eLSA dynamic programming algorithm (see Methods).

#### Permutation test

The statistical significance, the p-value, of LS score is evaluated using a permutation test. Briefly, eLSA randomly shuffles the components of the original time series and recalculates the LS score for the pairs. The p-value is approximated by the fraction of permutation scores that are larger (in absolute value) than the original score. Confidence interval for a given LS score is also found by bootstrapping from the replicated data. Finally, users can obtain significant eLSA association results by the combined use of p-value and FDR q-value thresholds as their filtering criteria.

#### Association network construction

Using only the significant associations, users can construct a partially directed association network. Generally, for two factors *X* and *Y*, if the time interval [*s*_1_,*t*_1_] in *X* and [*s*_2_,*t*_2_] in *Y* have the highest LS and *s*_1_ <*s*_2_, we can infer that *X* leads *Y*; in other words, *X* possibly activates *Y*. In network visualization software (*e.g.,* Cytoscape [24]), one can use arrows to directionally indicate these lead patterns (i.e., *X* to *Y*, if *X* leads *Y*; otherwise undirected, if no direction is inferred). One can also use lines to indicate association types (solid, if *X* is positively associated with *Y*; otherwise dashed). Following these rules, one can build a partially directed association network based on eLSA results.

### Microbial community data analysis

As an immediate application, we applied the eLSA pipeline to a set of real microbial community time series data. This San Pedro Ocean Time Series (SPOTs) dataset, originally reported in *Steele et al.*[2] and *Countway et al.*[25], was collected following a biological feature (i.e. the chlorophyll maximum depth) off the coast of Southern California. The bacterial community was analyzed using the ARISA [4] technique and the protistan community was analyzed using the T-RFLP [26] technique. The dataset is composed of monthly sampled data from September 2000 to March 2004, including 40 time points without replicates. We analyzed the dataset with a delay limit of 3 months and 1000 permutations to evaluate the statistical significance of the LSA score. In this dataset, the factor names, including the operational taxonomic units and environmental factors, are previously defined by *Steele et al. *[2].

First, we compared the performance of Pearson’s correlation coefficient (PCC) and eLSA analysis in identifying potential local and time-delayed associations. Restricting the significance threshold for the q-value *Q* ≤ 0.01 and the p-value *P* ≤ 0.01, 1643 pairs of significant associations with eLSA were identified, and among them only 293 (~18%) were discovered by PCC (see Table 2). Therefore, most significant associations found by eLSA would have been missed by PCC analysis in this case. The results are similar if we use less stringent criteria, i.e., *Q* ≤ 0.05 and *P* ≤ 0.05, where only 658 out of 2804 (~23%) eLSA significant associations were also found by PCC. We need to point out that, PCC also found some associations that were missed by eLSA. For example, with q-value *Q* ≤ 0.01 and the p-value *P* ≤ 0.01, PCC found 3237 significant associations and only 293 of them were found to be significant using eLSA. Therefore, eLSA is not a substitute but a complimentary approach to PCC, which specializes in finding local and possibly time-delayed associations. For a thorough analysis of a dataset, one should apply both approaches, which is why we also integrated PCC analysis into our software pipeline.

**Table 2:** Significant associations found in real datasets

		Found by eLSA	Found by PCC	Found by both	Found by eLSA	Found by PCC	Found by both
Dataset	# of factors	*P* ≤ 0.01	*P* ≤ 0.01	*P* ≤ 0.01	*P* ≤ 0.05	*P* ≤ 0.05	*P* ≤ 0.05
		*Q* ≤ 0.01	*Q* ≤ 0.01	*Q* ≤ 0.01	*Q* ≤ 0.05	*Q* ≤ 0.05	*Q* ≤ 0.05
Microbial	515	1643	3237	293	2804	4242	658
*C. elegans*	446	42532	56605	39114	57991	71799	54201

Numbers of significant associations found by the extended Local Similarity Analysis (eLSA) and Pearson’s Correlation Coefficient (PCC) by controlling both the p-value (*P*) and the q-value (*Q*). The p-values for eLSA were evaluated by permutations and p-values for PCC was calculated based on the *t*-distribution.

**Figure 3:**
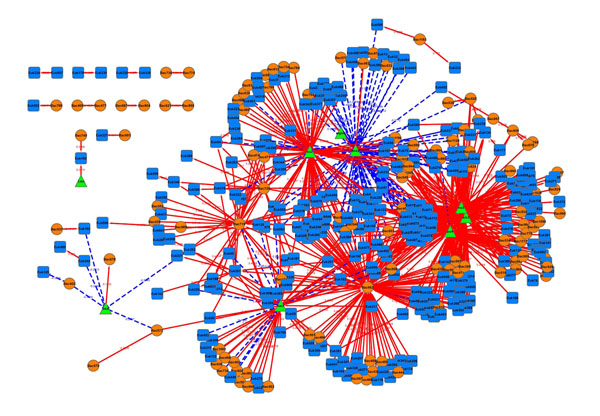
**Typical association network from the microbial community data.** Round- (brown), square- (blue) and triangle- (green) shaped nodes are bacteria, eukaryotes and environmental factors, respectively. Solid (red) edges are positively associated, while dashed (blue) edges are negatively associated. Arrow indicates the time-delay direction.

If we look at the top five positive and negative absolute highest LS scores from the unique associations (|*D*| ≤ 1) found by eLSA (*Q* ≤ 0.05 and *P* ≤ 0.05, see Table 3), we can see most of them are time-dependent associations, either time-shifted or within a subinterval. The majority of these are, in any case, beyond the capacity of PCC. In addition, eLSA provides more information about its findings. For example, in the table, *Bac609* and *Bac675* factors are associated with a shift of one and *Euk97* and *boxy* (oxygen) factors are best associated within a time interval of length 21 starting at time point 15 with no delay. This kind of additional information is not easily obtainable from the PCC analysis but very important for further functional analysis. For instance, we construct an association network using all above unique eLSA associations, as shown in Figure 3. The obtained network obviously reveals some interesting dynamics of the microbial community, such as the domination of positive directed associations, the existence of environmental factors as hubs that are associated with many other factors, (e.g. nutrients such as *NO*_2_, *PO*_4_, *SiO*_3_ and oxygen), and the existence of some highly connected clusters formed by certain bacteria or eukaryote groups.

**Table 3:** Top LS scores from the microbial community data

X	Y	LS	Xs	Ys	Len	D	P	PCC	Ppcc	Q	Qpcc
Euk239	Euk269	0.82	1	1	40	0	0	0.09	0.59	0.02	1.00
Bac609	Bac675	0.77	1	2	39	-1	0	0.14	0.41	0.00	1.00
Euk381	Euk462	0.77	1	1	40	0	0	0.44	0.00	0.02	0.11
Euk583	Bac989	0.68	2	1	39	1	0	0.30	0.06	0.02	0.73
Euk229	Euk339	0.57	1	2	39	-1	0	0.05	0.77	0.02	1.00
Euk97	boxy	-0.62	15	15	21	0	0	-0.42	0.01	0.00	0.17
Euk98	boxy	-0.62	15	15	21	0	0	-0.42	0.01	0.00	0.17
Euk109	boxy	-0.62	15	15	21	0	0	-0.42	0.01	0.00	0.17
Euk112	boxy	-0.62	15	15	21	0	0	-0.42	0.01	0.00	0.17
Euk116	boxy	-0.62	15	15	21	0	0	-0.42	0.01	0.00	0.17

The 5 positive and 5 negative highest absolute LS Scores from associations uniquely found by eLSA in the microbial community dataset. The columns in succession are X (first factor), Y (second factor), LS (Local Similarity score), Xs (start of the best alignment in the first sequence), Ys (start of the best alignment in the second sequence), Len (alignment length), D (shift of the second sequence compared to the first sequence, -: X is ahead of Y, + otherwise), P (p-value for the LS score, 0.00 stands for P<0.005), PCC (Pearson’s Correlation Coefficient), Ppcc (P-value for PCC), Q (q-value calculated for P, 0.00 stands for Q<0.005), Qpcc (q-value for Ppcc).

**Figure 4:**
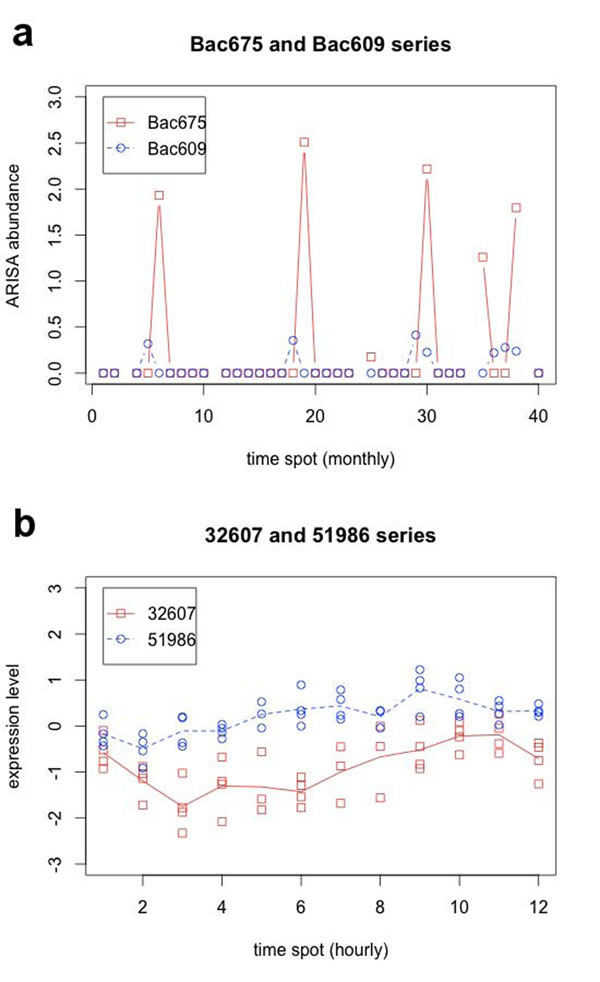
**Examples of real data association. a**. Shown are microbe group *Bac675* (red square) and *Bac609* (blue circle) ARISA abundance time series from the marine microbial community data analysis. Notice that there exists an almost regular yearly pattern where *Bac609* leads *Bac675* by one month in blooming time. **b**. Shown are gene *32607* (red square) and *51986* (blue circle) expression level time series from *C. elegans* gene expression data analysis. Notice that *51986* leads *32607* in expression level change throughout the time course.

Taking a closer look at one of the topmost ranked association: *Bac609* and *Bac675* (see Table 3), we found that they are closely following each other with a time shift of one month, where *Bac609* precedes *Bac675*. Further inspection suggests a yearly pattern that recurs with near regularity for this association, such that *Bac609* blooms in early springtime each year (time spots 6, 18 and 29 are February, January and March, respectively), and *Bac675* blooms one month later (see Figure 4a). From the binning definition in *Steele et al. *[2], *Bac609* is a *Bacteroidetes* group bacterium while *Bac675* is an undefined bacterium. Since these microbial groups are uncultured, this association as well as many others uniquely identified by eLSA provides new insight into their ecological role in the ocean surface waters. Notice there is an unexpected abundance jump at time spot 35 of the *Bac675* series. The reason for this outlier however is unknown to us. While such prominent time-delayed associations as the *Bac609* and *Bac675* are easily visible, we must caution that time-dependent associations could also be too subtle to be viewed directly. Thus, statistical significance can provide a much more reliable guideline.

### Gene expression data analysis

Although LSA had its roots grounded in microbial community analysis, the technique can be readily applied to other biological time series data, such as replicated gene expression time series data from microarray and RNA-Seq experiments [27-29]. Here we show an example of applying eLSA to the dauer exit gene expression profile time series data of 446 genes from a *C elegans* study. The result of the original study suggests that the 446 genes under investigation have similar kinetics in both the dauer exit and the L1 starvation time course [30]. Here we use the dauer exit time series data consisting of 12 hourly time spots, each with four replicates. We analyzed the dataset with a delay limit of 3 hours and with 1000 permutations and 100 bootstraps.

The results are summarized in Table 2. Comparing the *C. elegans* results to those of the microbial community, we see that gene-gene associations in this network are much denser, since a smaller number of genes end up with a much larger, rather than smaller, number of eLSA significant associations (e.g. 2804 versus 57991 for *Q* ≤ 0.05 and *P* ≤ 0.05, see Table 2). Also different is that about 93% of these associations are found by PCC analysis as well. The high congruence between PCC and eLSA analysis may be due to the fact that about 90% of the eLSA findings are without delays, which thus are also amenable to PCC analysis.

Because these genes do not change expression level in both dauer exit and L1 starvation conditions, they are considered as common feeding response genes [30]. However, it is not clear whether they are correlated with each other in expression profiles under the dauer exit condition. To study this, we combined all eLSA and PCC significant associations with *Q* ≤ 0.05 and *P* ≤ 0.05, and found the average degree of the resulting association network is around 169, while that of previous microbial community data is around 12. Such high average degree for *C. elegans* genes shows the high similarity of their expression profiles, which also reflects their intimate functional coordination along the process. Therefore, our result suggests those feeding response genes are likely to be co-expressed under the dauer exit condition.

**Figure 5:**
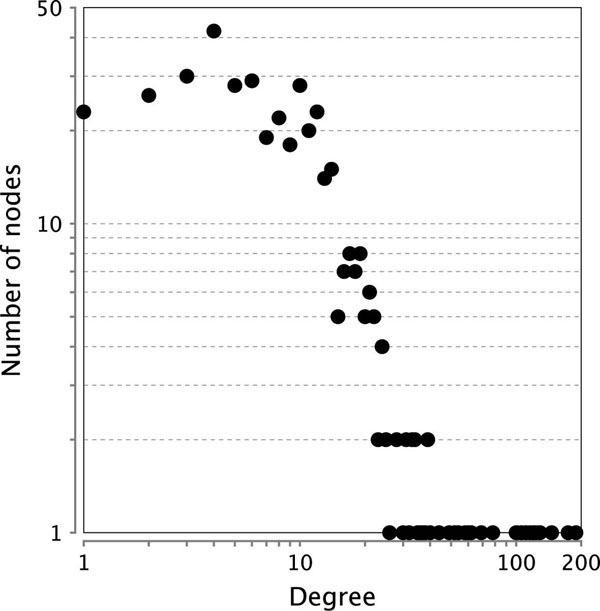
**Node degree distribution of associations in *C. elegans* analysis**. Shown is the node degree distribution of eLSA unique associations in C. elegans analysis. It shows a long-tail distribution with the maximum 189.

We next analyzed the unique eLSA associations. These associations form a dense association network themselves with a long-tailed degree distribution, as shown in Figure 5. While the degree distribution peaks at five, the most highly connected gene *48941* has a degree of 189. We also looked at the top 5 positive and 5 negative highest absolute LS scores unique associations by eLSA. Because replicates are available for this dataset, we are able to obtain the bootstrap confidence intervals for the LS score and they are given in Table 4. Interestingly, we found most of the top LS associations involve high degree nodes, such as genes *48941*(189), *29494*(129), *29504*(128), *27993*(116), *436287*(106), *32607*(58), and *51986*(52) (degree in parenthesis). These high degree nodes could be regulation hubs in the feeding response pathway. Here we show an example of time-delayed association of gene *32607* and gene *51986* in Figure 4b. In the figure, gene *51986* leads gene *32607* in expression profile change.

**Table 4:** Top LS scores from the *C. elegans* gene-expression data

X	Y	LS	lowCI	upCI	Xs	Ys	Len	D	P	PCC	Ppcc	Q	Qpcc
48087	27993	0.53	0.41	0.61	1	2	11	-1	0.00	0.56	0.06	0.00	0.01
32607	51986	0.52	0.41	0.61	2	1	10	1	0.01	0.51	0.09	0.00	0.01
29504	48087	0.52	0.40	0.61	2	1	11	1	0.00	0.41	0.18	0.00	0.03
23193	27993	0.51	0.41	0.59	1	2	11	-1	0.00	0.48	0.11	0.00	0.02
29494	30208	0.51	0.39	0.61	2	1	11	1	0.00	0.58	0.05	0.00	0.01
27993	53694	-0.55	-0.62	-0.44	2	1	11	1	0.00	-0.53	0.08	0.00	0.01
436287	53694	-0.54	-0.62	-0.44	2	1	11	1	0.01	-0.55	0.06	0.00	0.01
48941	53694	-0.52	-0.61	-0.42	2	1	11	1	0.00	-0.38	0.22	0.00	0.03
29494	22857	-0.52	-0.61	-0.41	2	1	11	1	0.00	-0.49	0.10	0.00	0.02
29494	436727	-0.52	-0.61	-0.40	2	1	11	1	0.01	-0.55	0.06	0.00	0.01

The 5 positive and 5 negative highest absolute LS Scores from the *C. elegans* gene expression dataset. The notations are the same as in Table 3 except lowCI (CI is lower bound) and upCI (CI is upper bound) in the 4^th^ and 5^th^ columns.

**Figure 6:**
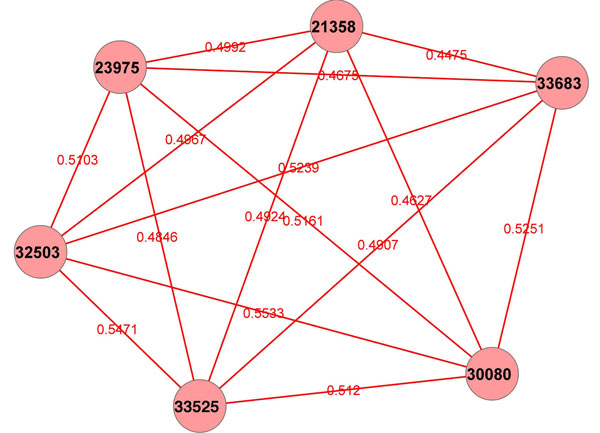
**Translation initiation factor associations in *C. elegans* analysis.** Shown is the association network of translation initiation factors learned from eLSA analysis. Solid (red) edges are positively associated. Edge labels are LS scores. The factors form a clique as expected.

We also analyzed all the eLSA associations together, including both unique and non-unique eLSA findings. Though most of the genes are still hypothetical protein coding genes, we do find a group of eukaryotic initiation factors: *30080*(eIF-3E), *33683*(eIF-3K), *21358*(eIF-3D), *33525*(eIF-4E), *32503*(eIF-1A) and *23975*(eIF-2B) in the 446 selected genes. This is as expected because both L1 starvation recovery and dauer exit will increase translation activities and result in high expression level of these genes. In addition, in the translation process, these factors work closely together to form different translation related complexes [31], so their expression levels should be highly correlated with each other. Actually, if we check the associations found by eLSA, we do see these factors form a clique together with all edges being positive associations and statistically significant (see Figure 6). The coherence of the eLSA finding and our biological knowledge shows that eLSA associations do reveal true associations within the biological system. However, as the majority of genes are still hypothetical, a thorough examination for true functional discoveries will require biological experiments.

## Discussion and conclusions

The eLSA technique extends LSA to time series data with replicates. This will help investigators better utilize the available information from their sample replicates and assist them in more effective and reliable hypothesis generation of time-dependent associations. In addition, a bootstrap framework is developed to estimate the confidence interval for the LS score. We also provided flexible missing value options and integrated efficient multiple testing control methods for the new eLSA technique. Using the microbial community and gene expression datasets, we demonstrated that eLSA uniquely captures additional time-dependent associations, including local and time-delayed association patterns, when compared to ordinary correlation methods, such as PCC. In this paper, we described the applications of our method with the time series data. Actually, the eLSA can be applied to any type of data with some gradients, including the response to different levels of treatments, temperature, humidity, or spatial distributions.

Currently, we use permutation test to assess the statistical significance of LS scores and bootstrap re-sampling to estimate the confidence interval of LS score. Both the permutation test and bootstrap methods are time consuming if high precise determination of statistical significance or confidence interval is desired. Theoretical developments on the distribution of the LS score are needed to eliminate or mitigate the computational burden required for these processes, and would be interesting topics for future studies. There is also a minimum sample number requirement for eLSA analysis. We suggest the sample number to be greater than 5+*D*, where *D* is the desired delay limit, since shifting and trimming by eLSA will further reduce the effective sample number and result in lower statistical power.

**Figure 7:**
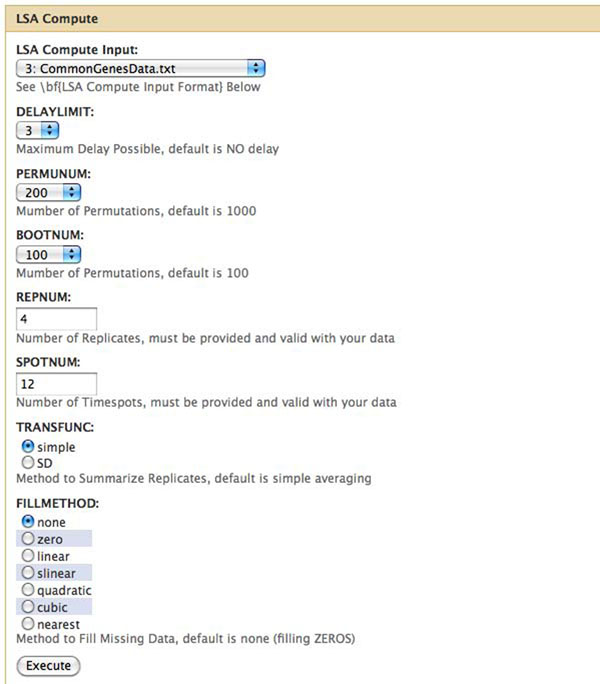
**Submission interface for the LSA web service.** Upon submission, the job will perform eLSA analysis on the ‘CommonGenesData’ dataset (12 time spots and 4 replicates) with 200 permutations and 100 bootstraps within a delay limit of 3 units. In addition, by specification, it will use ‘simple’ averaging to summarize replicates and, by designating ‘none’, it will disregard the missing values.

Finally, we implemented the eLSA technique and analysis pipeline into an Open Source C++ extension to Python with many new features. Specifically, the pipeline streamlines data normalization, local similarity scoring, permutation testing and network construction. As shown in Figure 7, we also provide a Galaxy web framework-based version [22] of the eLSA pipeline. This eLSA service features customized workflow, history and data sharing. In addition, we integrated Cytoscape [23] Java Web Start technology so that the association network generated by eLSA can be immediately visualized. Based on these efforts, we anticipate that our novel eLSA methodology, as implemented by the newly developed pipeline software, will significantly assist researchers requiring systematic discovery of time-dependent associations. More information about the software and web services is available from the eLSA homepage at http://meta.usc.edu/softs/lsa.

## Authors' contributions

LCX, JAS, JAC, ZGC, SLS, JJV, JAF, FS designed the study. LCX, ZGC, JAF and FS developed the methods. LCX, JAS, JAC developed and tested the software. LCX, JAS and JAC collected and analyzed the data. LCX, JAS, JAC, ZGC, JAF and FS wrote the paper.

## Competing interests

The authors declare that they have no competing interests.
